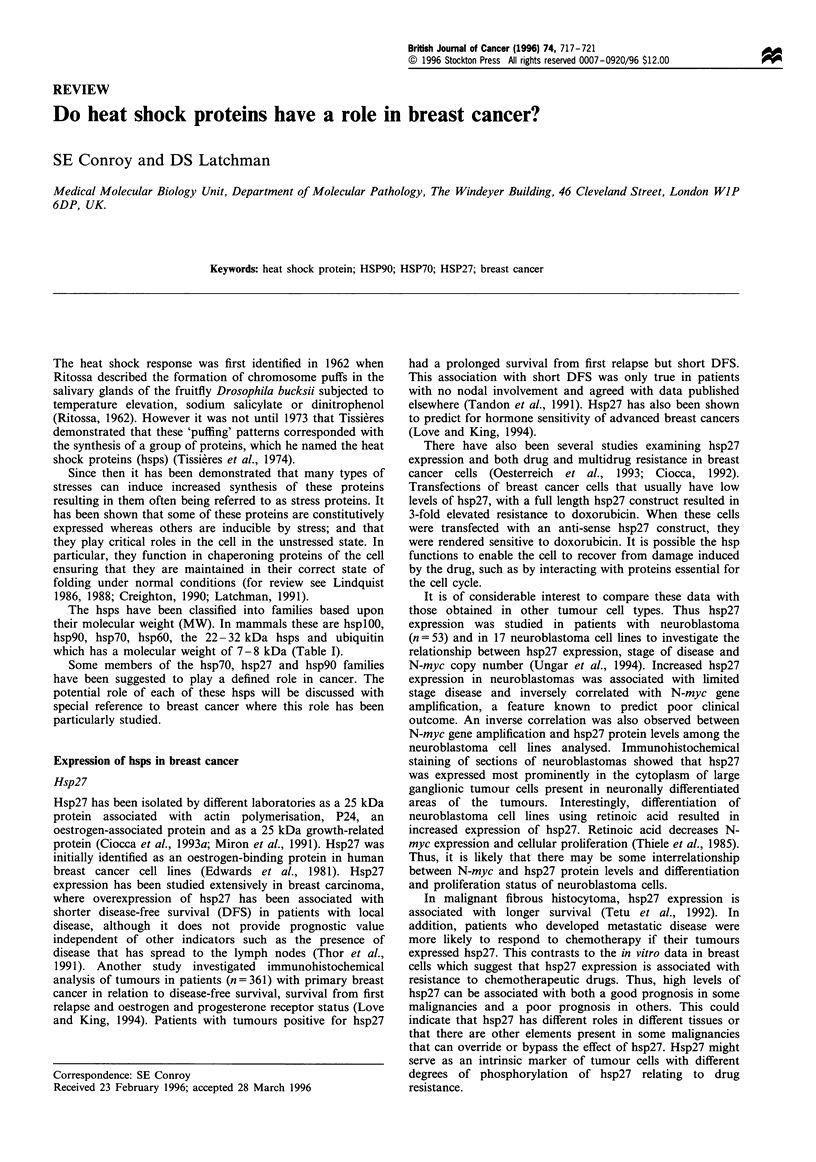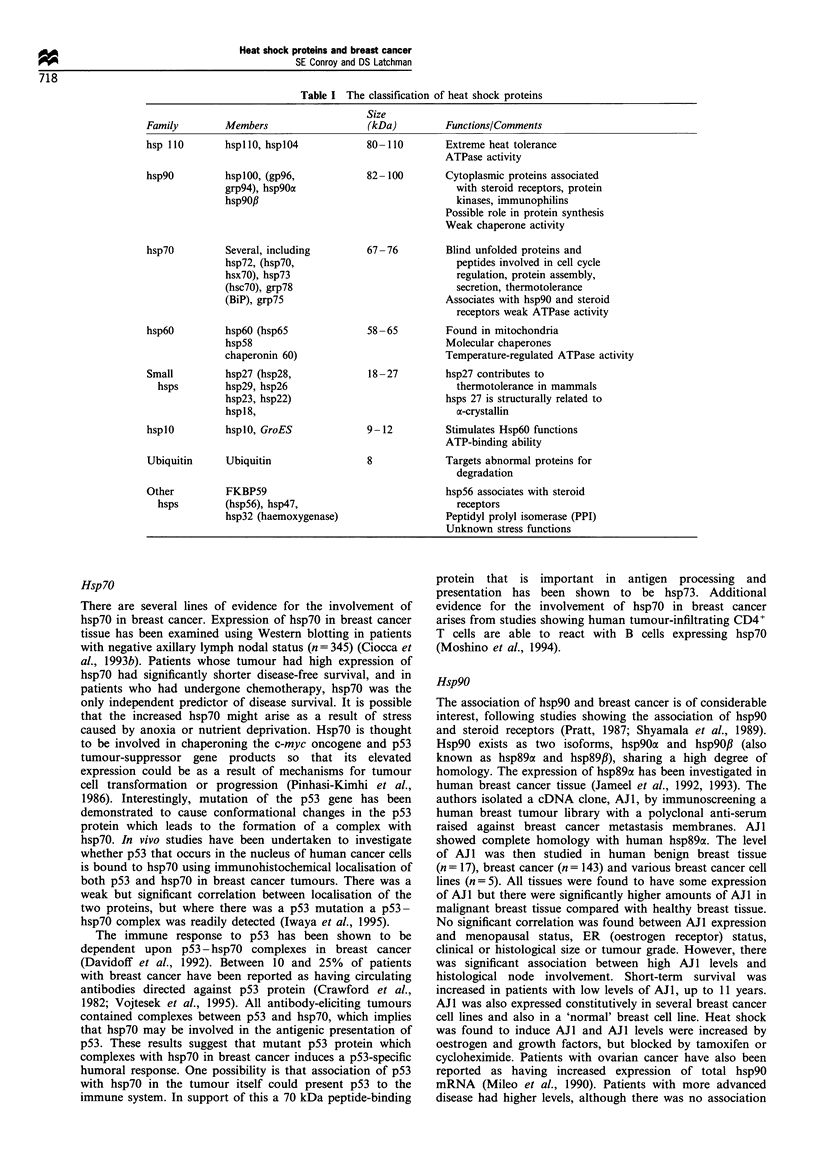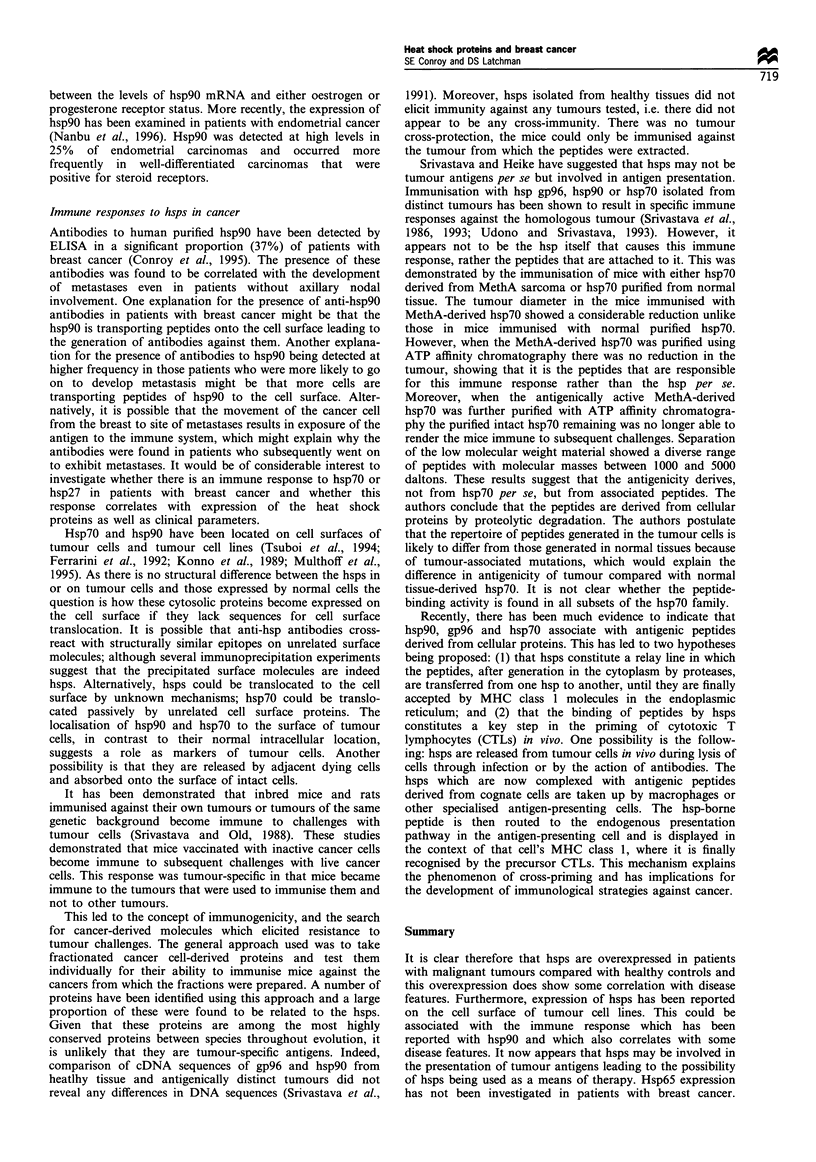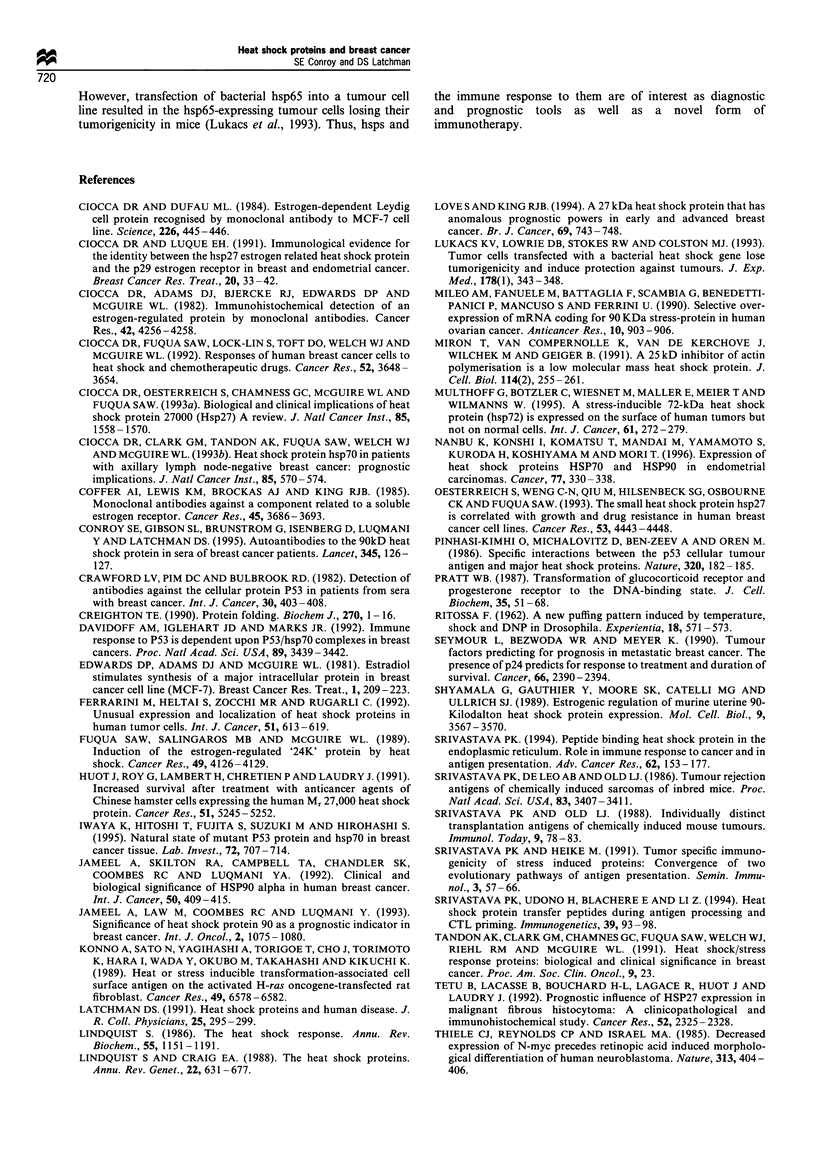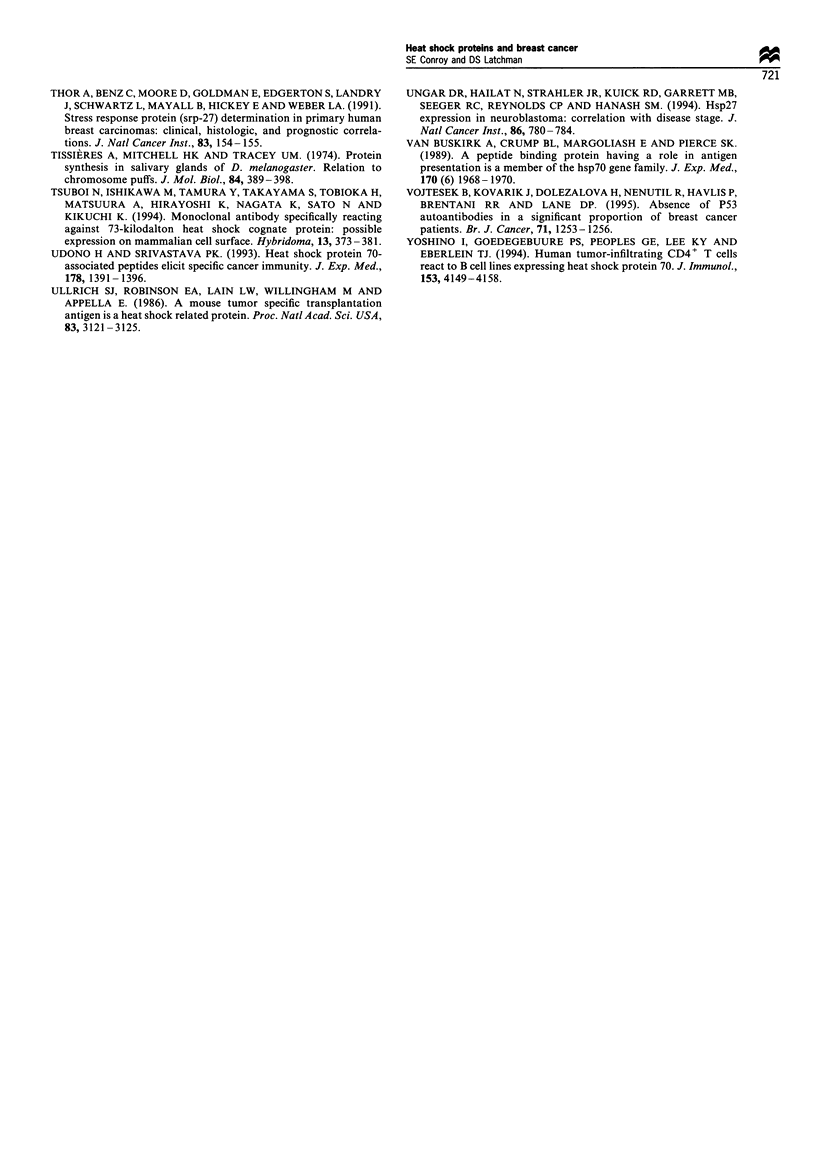# Do heat shock proteins have a role in breast cancer?

**DOI:** 10.1038/bjc.1996.427

**Published:** 1996-09

**Authors:** S. E. Conroy, D. S. Latchman

**Affiliations:** Medical Molecular Biology Unit, Department of Molecular Pathology, London, UK.

## Abstract

It is clear therefore that hsps are overexpressed in patients with malignant tumours compared with healthy controls and this overexpression does show some correlation with disease features. Furthermore, expression of hsps has been reported on the cell surface of tumour cell lines. This could be associated with the immune response which has been reported with hsp90 and which also correlates with some disease features. It now appears that hsps may be involved in the presentation of tumour antigens leading to the possibility of hsps being used as a means of therapy. Hsp65 expression has not been investigated in patients with breast cancer. However, transfection of bacterial hsp65 into a tumour cell line resulted in the hsp65-expressing tumour cells losing their tumorigenicity in mice (Lukacs et al., 1993). Thus, hsps and the immune response to them are of interest as diagnostic and prognostic tools as well as a novel form of immunotherapy.


British Journal of Cancer (1996) 74, 717-721
© 1996 Stockton Press All rights reserved 0007-0920/96 $12.00

**REVIEW**

### Do heat shock proteins have a role in breast cancer?

SE Conroy and DS Latchman

*Medical Molecular Biology Unit, Department of Molecular Pathology, The Windeyer Building, 46 Cleveland Street, London W1P 6DP, UK.*

**Keywords:** heat shock protein; HSP90; HSP70; HSP27; breast cancer

The heat shock response was first identified in 1962 when Ritossa described the formation of chromosome puffs in the salivary glands of the fruitfly *Drosophila bucksii* subjected to temperature elevation, sodium salicylate or dinitrophenol (Ritossa, 1962). However it was not until 1973 that Tissières demonstrated that these 'puffing' patterns corresponded with the synthesis of a group of proteins, which he named the heat shock proteins (hsps) (Tissières *et al.*, 1974).

Since then it has been demonstrated that many types of stresses can induce increased synthesis of these proteins resulting in them often being referred to as stress proteins. It has been shown that some of these proteins are constitutively expressed whereas others are inducible by stress; and that they play critical roles in the cell in the unstressed state. In particular, they function in chaperoning proteins of the cell ensuring that they are maintained in their correct state of folding under normal conditions (for review see Lindquist 1986, 1988; Creighton, 1990; Latchman, 1991).

The hsps have been classified into families based upon their molecular weight (MW). In mammals these are hsp100, hsp90, hsp70, hsp60, the 22-32 kDa hsps and ubiquitin which has a molecular weight of 7-8 kDa (Table I).

Some members of the hsp70, hsp27 and hsp90 families have been suggested to play a defined role in cancer. The potential role of each of these hsps will be discussed with special reference to breast cancer where this role has been particularly studied.

### Expression of hsps in breast cancer

### *Hsp27*

Hsp27 has been isolated by different laboratories as a 25 kDa protein associated with actin polymerisation, P24, an oestrogen-associated protein and as a 25 kDa growth-related protein (Ciocca *et al.*, 1993*a*; Miron *et al.*, 1991). Hsp27 was initially identified as an oestrogen-binding protein in human breast cancer cell lines (Edwards *et al.*, 1981). Hsp27 expression has been studied extensively in breast carcinoma, where overexpression of hsp27 has been associated with shorter disease-free survival (DFS) in patients with local disease, although it does not provide prognostic value independent of other indicators such as the presence of disease that has spread to the lymph nodes (Thor *et al.*, 1991). Another study investigated immunohistochemical analysis of tumours in patients ($n = 361$) with primary breast cancer in relation to disease-free survival, survival from first relapse and oestrogen and progesterone receptor status (Love and King, 1994). Patients with tumours positive for hsp27 had a prolonged survival from first relapse but short DFS. This association with short DFS was only true in patients with no nodal involvement and agreed with data published elsewhere (Tandon *et al.*, 1991). Hsp27 has also been shown to predict for hormone sensitivity of advanced breast cancers (Love and King, 1994).

There have also been several studies examining hsp27 expression and both drug and multidrug resistance in breast cancer cells (Oesterreich *et al.*, 1993; Ciocca, 1992). Transfections of breast cancer cells that usually have low levels of hsp27, with a full length hsp27 construct resulted in 3-fold elevated resistance to doxorubicin. When these cells were transfected with an anti-sense hsp27 construct, they were rendered sensitive to doxorubicin. It is possible the hsp functions to enable the cell to recover from damage induced by the drug, such as by interacting with proteins essential for the cell cycle.

It is of considerable interest to compare these data with those obtained in other tumour cell types. Thus hsp27 expression was studied in patients with neuroblastoma ($n = 53$) and in 17 neuroblastoma cell lines to investigate the relationship between hsp27 expression, stage of disease and N-*myc* copy number (Ungar *et al.*, 1994). Increased hsp27 expression in neuroblastomas was associated with limited stage disease and inversely correlated with N-*myc* gene amplification, a feature known to predict poor clinical outcome. An inverse correlation was also observed between N-*myc* gene amplification and hsp27 protein levels among the neuroblastoma cell lines analysed. Immunohistochemical staining of sections of neuroblastomas showed that hsp27 was expressed most prominently in the cytoplasm of large ganglionic tumour cells present in neuronally differentiated areas of the tumours. Interestingly, differentiation of neuroblastoma cell lines using retinoic acid resulted in increased expression of hsp27. Retinoic acid decreases N-*myc* expression and cellular proliferation (Thiele *et al.*, 1985). Thus, it is likely that there may be some interrelationship between N-*myc* and hsp27 protein levels and differentiation and proliferation status of neuroblastoma cells.

In malignant fibrous histocytoma, hsp27 expression is associated with longer survival (Tetu *et al.*, 1992). In addition, patients who developed metastatic disease were more likely to respond to chemotherapy if their tumours expressed hsp27. This contrasts to the *in vitro* data in breast cells which suggest that hsp27 expression is associated with resistance to chemotherapeutic drugs. Thus, high levels of hsp27 can be associated with both a good prognosis in some malignancies and a poor prognosis in others. This could indicate that hsp27 has different roles in different tissues or that there are other elements present in some malignancies that can override or bypass the effect of hsp27. Hsp27 might serve as an intrinsic marker of tumour cells with different degrees of phosphorylation of hsp27 relating to drug resistance.

Correspondence: SE Conroy
Received 23 February 1996; accepted 28 March 1996

Table I The classification of heat shock proteins

| Family | Members | Size (kDa) | Functions/Comments |
|---|---|---|---|
| hsp 110 | hsp110, hsp104 | 80–110 | Extreme heat tolerance<br>ATPase activity |
| hsp90 | hsp100, (gp96, grp94), hsp90α hsp90β | 82–100 | Cytoplasmic proteins associated with steroid receptors, protein kinases, immunophilins<br>Possible role in protein synthesis<br>Weak chaperone activity |
| hsp70 | Several, including hsp72, (hsp70, hsx70), hsp73 (hsc70), grp78 (BiP), grp75 | 67–76 | Blind unfolded proteins and peptides involved in cell cycle regulation, protein assembly, secretion, thermotolerance<br>Associates with hsp90 and steroid receptors weak ATPase activity |
| hsp60 | hsp60 (hsp65 hsp58 chaperonin 60) | 58–65 | Found in mitochondria<br>Molecular chaperones<br>Temperature-regulated ATPase activity |
| Small hsps | hsp27 (hsp28, hsp29, hsp26 hsp23, hsp22) hsp18, | 18–27 | hsp27 contributes to thermotolerance in mammals<br>hsps 27 is structurally related to α-crystallin |
| hsp10 | hsp10, *GroES* | 9–12 | Stimulates Hsp60 functions<br>ATP-binding ability |
| Ubiquitin | Ubiquitin | 8 | Targets abnormal proteins for degradation |
| Other hsps | FKBP59 (hsp56), hsp47, hsp32 (haemoxygenase) | | hsp56 associates with steroid receptors<br>Peptidyl prolyl isomerase (PPI)<br>Unknown stress functions |

### *Hsp70*

There are several lines of evidence for the involvement of hsp70 in breast cancer. Expression of hsp70 in breast cancer tissue has been examined using Western blotting in patients with negative axillary lymph nodal status ($n=345$) (Ciocca *et al.*, 1993*b*). Patients whose tumour had high expression of hsp70 had significantly shorter disease-free survival, and in patients who had undergone chemotherapy, hsp70 was the only independent predictor of disease survival. It is possible that the increased hsp70 might arise as a result of stress caused by anoxia or nutrient deprivation. Hsp70 is thought to be involved in chaperoning the c-*myc* oncogene and p53 tumour-suppressor gene products so that its elevated expression could be as a result of mechanisms for tumour cell transformation or progression (Pinhasi-Kimhi *et al.*, 1986). Interestingly, mutation of the p53 gene has been demonstrated to cause conformational changes in the p53 protein which leads to the formation of a complex with hsp70. *In vivo* studies have been undertaken to investigate whether p53 that occurs in the nucleus of human cancer cells is bound to hsp70 using immunohistochemical localisation of both p53 and hsp70 in breast cancer tumours. There was a weak but significant correlation between localisation of the two proteins, but where there was a p53 mutation a p53–hsp70 complex was readily detected (Iwaya *et al.*, 1995).

The immune response to p53 has been shown to be dependent upon p53–hsp70 complexes in breast cancer (Davidoff *et al.*, 1992). Between 10 and 25% of patients with breast cancer have been reported as having circulating antibodies directed against p53 protein (Crawford *et al.*, 1982; Vojtesek *et al.*, 1995). All antibody-eliciting tumours contained complexes between p53 and hsp70, which implies that hsp70 may be involved in the antigenic presentation of p53. These results suggest that mutant p53 protein which complexes with hsp70 in breast cancer induces a p53-specific humoral response. One possibility is that association of p53 with hsp70 in the tumour itself could present p53 to the immune system. In support of this a 70 kDa peptide-binding protein that is important in antigen processing and presentation has been shown to be hsp73. Additional evidence for the involvement of hsp70 in breast cancer arises from studies showing human tumour-infiltrating $CD4^+$ T cells are able to react with B cells expressing hsp70 (Moshino *et al.*, 1994).

### *Hsp90*

The association of hsp90 and breast cancer is of considerable interest, following studies showing the association of hsp90 and steroid receptors (Pratt, 1987; Shyamala *et al.*, 1989). Hsp90 exists as two isoforms, hsp90α and hsp90β (also known as hsp89α and hsp89β), sharing a high degree of homology. The expression of hsp89α has been investigated in human breast cancer tissue (Jameel *et al.*, 1992, 1993). The authors isolated a cDNA clone, AJ1, by immunoscreening a human breast tumour library with a polyclonal anti-serum raised against breast cancer metastasis membranes. AJ1 showed complete homology with human hsp89α. The level of AJ1 was then studied in human benign breast tissue ($n=17$), breast cancer ($n=143$) and various breast cancer cell lines ($n=5$). All tissues were found to have some expression of AJ1 but there were significantly higher amounts of AJ1 in malignant breast tissue compared with healthy breast tissue. No significant correlation was found between AJ1 expression and menopausal status, ER (oestrogen receptor) status, clinical or histological size or tumour grade. However, there was significant association between high AJ1 levels and histological node involvement. Short-term survival was increased in patients with low levels of AJ1, up to 11 years. AJ1 was also expressed constitutively in several breast cancer cell lines and also in a 'normal' breast cell line. Heat shock was found to induce AJ1 and AJ1 levels were increased by oestrogen and growth factors, but blocked by tamoxifen or cycloheximide. Patients with ovarian cancer have also been reported as having increased expression of total hsp90 mRNA (Mileo *et al.*, 1990). Patients with more advanced disease had higher levels, although there was no association between the levels of hsp90 mRNA and either oestrogen or progesterone receptor status. More recently, the expression of hsp90 has been examined in patients with endometrial cancer (Nanbu *et al.*, 1996). Hsp90 was detected at high levels in 25% of endometrial carcinomas and occurred more frequently in well-differentiated carcinomas that were positive for steroid receptors.

### *Immune responses to hsps in cancer*

Antibodies to human purified hsp90 have been detected by ELISA in a significant proportion (37%) of patients with breast cancer (Conroy *et al.*, 1995). The presence of these antibodies was found to be correlated with the development of metastases even in patients without axillary nodal involvement. One explanation for the presence of anti-hsp90 antibodies in patients with breast cancer might be that the hsp90 is transporting peptides onto the cell surface leading to the generation of antibodies against them. Another explanation for the presence of antibodies to hsp90 being detected at higher frequency in those patients who were more likely to go on to develop metastasis might be that more cells are transporting peptides of hsp90 to the cell surface. Alternatively, it is possible that the movement of the cancer cell from the breast to site of metastases results in exposure of the antigen to the immune system, which might explain why the antibodies were found in patients who subsequently went on to exhibit metastases. It would be of considerable interest to investigate whether there is an immune response to hsp70 or hsp27 in patients with breast cancer and whether this response correlates with expression of the heat shock proteins as well as clinical parameters.

Hsp70 and hsp90 have been located on cell surfaces of tumour cells and tumour cell lines (Tsuboi *et al.*, 1994; Ferrarini *et al.*, 1992; Konno *et al.*, 1989; Multhoff *et al.*, 1995). As there is no structural difference between the hsps in or on tumour cells and those expressed by normal cells the question is how these cytosolic proteins become expressed on the cell surface if they lack sequences for cell surface translocation. It is possible that anti-hsp antibodies cross-react with structurally similar epitopes on unrelated surface molecules; although several immunoprecipitation experiments suggest that the precipitated surface molecules are indeed hsps. Alternatively, hsps could be translocated to the cell surface by unknown mechanisms; hsp70 could be translocated passively by unrelated cell surface proteins. The localisation of hsp90 and hsp70 to the surface of tumour cells, in contrast to their normal intracellular location, suggests a role as markers of tumour cells. Another possibility is that they are released by adjacent dying cells and absorbed onto the surface of intact cells.

It has been demonstrated that inbred mice and rats immunised against their own tumours or tumours of the same genetic background become immune to challenges with tumour cells (Srivastava and Old, 1988). These studies demonstrated that mice vaccinated with inactive cancer cells become immune to subsequent challenges with live cancer cells. This response was tumour-specific in that mice became immune to the tumours that were used to immunise them and not to other tumours.

This led to the concept of immunogenicity, and the search for cancer-derived molecules which elicited resistance to tumour challenges. The general approach used was to take fractionated cancer cell-derived proteins and test them individually for their ability to immunise mice against the cancers from which the fractions were prepared. A number of proteins have been identified using this approach and a large proportion of these were found to be related to the hsps. Given that these proteins are among the most highly conserved proteins between species throughout evolution, it is unlikely that they are tumour-specific antigens. Indeed, comparison of cDNA sequences of gp96 and hsp90 from heatlhy tissue and antigenically distinct tumours did not reveal any differences in DNA sequences (Srivastava *et al.*, 1991). Moreover, hsps isolated from healthy tissues did not elicit immunity against any tumours tested, i.e. there did not appear to be any cross-immunity. There was no tumour cross-protection, the mice could only be immunised against the tumour from which the peptides were extracted.

Srivastava and Heike have suggested that hsps may not be tumour antigens *per se* but involved in antigen presentation. Immunisation with hsp gp96, hsp90 or hsp70 isolated from distinct tumours has been shown to result in specific immune responses against the homologous tumour (Srivastava *et al.*, 1986, 1993; Udono and Srivastava, 1993). However, it appears not to be the hsp itself that causes this immune response, rather the peptides that are attached to it. This was demonstrated by the immunisation of mice with either hsp70 derived from MethA sarcoma or hsp70 purified from normal tissue. The tumour diameter in the mice immunised with MethA-derived hsp70 showed a considerable reduction unlike those in mice immunised with normal purified hsp70. However, when the MethA-derived hsp70 was purified using ATP affinity chromatography there was no reduction in the tumour, showing that it is the peptides that are responsible for this immune response rather than the hsp *per se*. Moreover, when the antigenically active MethA-derived hsp70 was further purified with ATP affinity chromatography the purified intact hsp70 remaining was no longer able to render the mice immune to subsequent challenges. Separation of the low molecular weight material showed a diverse range of peptides with molecular masses between 1000 and 5000 daltons. These results suggest that the antigenicity derives, not from hsp70 *per se*, but from associated peptides. The authors conclude that the peptides are derived from cellular proteins by proteolytic degradation. The authors postulate that the repertoire of peptides generated in the tumour cells is likely to differ from those generated in normal tissues because of tumour-associated mutations, which would explain the difference in antigenicity of tumour compared with normal tissue-derived hsp70. It is not clear whether the peptide-binding activity is found in all subsets of the hsp70 family.

Recently, there has been much evidence to indicate that hsp90, gp96 and hsp70 associate with antigenic peptides derived from cellular proteins. This has led to two hypotheses being proposed: (1) that hsps constitute a relay line in which the peptides, after generation in the cytoplasm by proteases, are transferred from one hsp to another, until they are finally accepted by MHC class 1 molecules in the endoplasmic reticulum; and (2) that the binding of peptides by hsps constitutes a key step in the priming of cytotoxic T lymphocytes (CTLs) *in vivo*. One possibility is the following: hsps are released from tumour cells *in vivo* during lysis of cells through infection or by the action of antibodies. The hsps which are now complexed with antigenic peptides derived from cognate cells are taken up by macrophages or other specialised antigen-presenting cells. The hsp-borne peptide is then routed to the endogenous presentation pathway in the antigen-presenting cell and is displayed in the context of that cell's MHC class 1, where it is finally recognised by the precursor CTLs. This mechanism explains the phenomenon of cross-priming and has implications for the development of immunological strategies against cancer.

### Summary

It is clear therefore that hsps are overexpressed in patients with malignant tumours compared with healthy controls and this overexpression does show some correlation with disease features. Furthermore, expression of hsps has been reported on the cell surface of tumour cell lines. This could be associated with the immune response which has been reported with hsp90 and which also correlates with some disease features. It now appears that hsps may be involved in the presentation of tumour antigens leading to the possibility of hsps being used as a means of therapy. Hsp65 expression has not been investigated in patients with breast cancer.

However, transfection of bacterial hsp65 into a tumour cell line resulted in the hsp65-expressing tumour cells losing their tumorigenicity in mice (Lukacs *et al.*, 1993). Thus, hsps and the immune response to them are of interest as diagnostic and prognostic tools as well as a novel form of immunotherapy.

### References

CIOCCA DR AND DUFAU ML. (1984). Estrogen-dependent Leydig cell protein recognised by monoclonal antibody to MCF-7 cell line. *Science*, **226**, 445–446.

CIOCCA DR AND LUQUE EH. (1991). Immunological evidence for the identity between the hsp27 estrogen related heat shock protein and the p29 estrogen receptor in breast and endometrial cancer. *Breast Cancer Res. Treat.*, **20**, 33–42.

CIOCCA DR, ADAMS DJ, BJERCKE RJ, EDWARDS DP AND MCGUIRE WL. (1982). Immunohistochemical detection of an estrogen-regulated protein by monoclonal antibodies. Cancer Res., **42**, 4256–4258.

CIOCCA DR, FUQUA SAW, LOCK-LIN S, TOFT DO, WELCH WJ AND MCGUIRE WL. (1992). Responses of human breast cancer cells to heat shock and chemotherapeutic drugs. *Cancer Res.*, **52**, 3648–3654.

CIOCCA DR, OESTERREICH S, CHAMNESS GC, MCGUIRE WL AND FUQUA SAW. (1993*a*). Biological and clinical implications of heat shock protein 27000 (Hsp27) A review. *J. Natl Cancer Inst.*, **85**, 1558–1570.

CIOCCA DR, CLARK GM, TANDON AK, FUQUA SAW, WELCH WJ AND MCGUIRE WL. (1993*b*). Heat shock protein hsp70 in patients with axillary lymph node-negative breast cancer: prognostic implications. *J. Natl Cancer Inst.*, **85**, 570–574.

COFFER AI, LEWIS KM, BROCKAS AJ AND KING RJB. (1985). Monoclonal antibodies against a component related to a soluble estrogen receptor. *Cancer Res.*, **45**, 3686–3693.

CONROY SE, GIBSON SL, BRUNSTROM G, ISENBERG D, LUQMANI Y AND LATCHMAN DS. (1995). Autoantibodies to the 90kD heat shock protein in sera of breast cancer patients. *Lancet*, **345**, 126–127.

CRAWFORD LV, PIM DC AND BULBROOK RD. (1982). Detection of antibodies against the cellular protein P53 in patients from sera with breast cancer. *Int. J. Cancer*, **30**, 403–408.

CREIGHTON TE. (1990). Protein folding. *Biochem J.*, **270**, 1–16.

DAVIDOFF AM, IGLEHART JD AND MARKS JR. (1992). Immune response to P53 is dependent upon P53/hsp70 complexes in breast cancers. *Proc. Natl Acad. Sci. USA*, **89**, 3439–3442.

EDWARDS DP, ADAMS DJ AND MCGUIRE WL. (1981). Estradiol stimulates synthesis of a major intracellular protein in breast cancer cell line (MCF-7). Breast Cancer Res. Treat., **1**, 209–223.

FERRARINI M, HELTAI S, ZOCCHI MR AND RUGARLI C. (1992). Unusual expression and localization of heat shock proteins in human tumor cells. *Int. J. Cancer*, **51**, 613–619.

FUQUA SAW, SALINGAROS MB AND MCGUIRE WL. (1989). Induction of the estrogen-regulated '24K' protein by heat shock. *Cancer Res.*, **49**, 4126–4129.

HUOT J, ROY G, LAMBERT H, CHRETIEN P AND LAUDRY J. (1991). Increased survival after treatment with anticancer agents of Chinese hamster cells expressing the human $M_r$ 27,000 heat shock protein. *Cancer Res.*, **51**, 5245–5252.

IWAYA K, HITOSHI T, FUJITA S, SUZUKI M AND HIROHASHI S. (1995). Natural state of mutant P53 protein and hsp70 in breast cancer tissue. *Lab. Invest.*, **72**, 707–714.

JAMEEL A, SKILTON RA, CAMPBELL TA, CHANDLER SK, COOMBES RC AND LUQMANI YA. (1992). Clinical and biological significance of HSP90 alpha in human breast cancer. *Int. J. Cancer*, **50**, 409–415.

JAMEEL A, LAW M, COOMBES RC AND LUQMANI Y. (1993). Significance of heat shock protein 90 as a prognostic indicator in breast cancer. *Int. J. Oncol.*, **2**, 1075–1080.

KONNO A, SATO N, YAGIHASHI A, TORIGOE T, CHO J, TORIMOTO K, HARA I, WADA Y, OKUBO M, TAKAHASHI AND KIKUCHI K. (1989). Heat or stress inducible transformation-associated cell surface antigen on the activated H-*ras* oncogene-transfected rat fibroblast. *Cancer Res.*, **49**, 6578–6582.

LATCHMAN DS. (1991). Heat shock proteins and human disease. *J. R. Coll. Physicians*, **25**, 295–299.

LINDQUIST S. (1986). The heat shock response. *Annu. Rev. Biochem.*, **55**, 1151–1191.

LINDQUIST S AND CRAIG EA. (1988). The heat shock proteins. *Annu. Rev. Genet.*, **22**, 631–677.

LOVE S AND KING RJB. (1994). A 27 kDa heat shock protein that has anomalous prognostic powers in early and advanced breast cancer. *Br. J. Cancer*, **69**, 743–748.

LUKACS KV, LOWRIE DB, STOKES RW AND COLSTON MJ. (1993). Tumor cells transfected with a bacterial heat shock gene lose tumorigenicity and induce protection against tumours. *J. Exp. Med.*, **178**(1), 343–348.

MILEO AM, FANUELE M, BATTAGLIA F, SCAMBIA G, BENEDETTI-PANICI P, MANCUSO S AND FERRINI U. (1990). Selective over-expression of mRNA coding for 90 KDa stress-protein in human ovarian cancer. *Anticancer Res.*, **10**, 903–906.

MIRON T, VAN COMPERNOLLE K, VAN DE KERCHOVE J, WILCHEK M AND GEIGER B. (1991). A 25 kD inhibitor of actin polymerisation is a low molecular mass heat shock protein. *J. Cell. Biol.* **114**(2), 255–261.

MULTHOFF G, BOTZLER C, WIESNET M, MALLER E, MEIER T AND WILMANNS W. (1995). A stress-inducible 72-kDa heat shock protein (hsp72) is expressed on the surface of human tumors but not on normal cells. *Int. J. Cancer*, **61**, 272–279.

NANBU K, KONSHI I, KOMATSU T, MANDAI M, YAMAMOTO S, KURODA H, KOSHIYAMA M AND MORI T. (1996). Expression of heat shock proteins HSP70 and HSP90 in endometrial carcinomas. *Cancer*, **77**, 330–338.

OESTERREICH S, WENG C-N, QIU M, HILSENBECK SG, OSBOURNE CK AND FUQUA SAW. (1993). The small heat shock protein hsp27 is correlated with growth and drug resistance in human breast cancer cell lines. *Cancer Res.*, **53**, 4443–4448.

PINHASI-KIMHI O, MICHALOVITZ D, BEN-ZEEV A AND OREN M. (1986). Specific interactions between the p53 cellular tumour antigen and major heat shock proteins. *Nature*, **320**, 182–185.

PRATT WB. (1987). Transformation of glucocorticoid receptor and progesterone receptor to the DNA-binding state. *J. Cell. Biochem*, **35**, 51–68.

RITOSSA F. (1962). A new puffing pattern induced by temperature, shock and DNP in Drosophila. *Experientia*, **18**, 571–573.

SEYMOUR L, BEZWODA WR AND MEYER K. (1990). Tumour factors predicting for prognosis in metastatic breast cancer. The presence of p24 predicts for response to treatment and duration of survival. *Cancer*, **66**, 2390–2394.

SHYAMALA G, GAUTHIER Y, MOORE SK, CATELLI MG AND ULLRICH SJ. (1989). Estrogenic regulation of murine uterine 90-Kilodalton heat shock protein expression. *Mol. Cell. Biol.*, **9**, 3567–3570.

SRIVASTAVA PK. (1994). Peptide binding heat shock protein in the endoplasmic reticulum. Role in immune response to cancer and in antigen presentation. *Adv. Cancer Res.*, **62**, 153–177.

SRIVASTAVA PK, DE LEO AB AND OLD LJ. (1986). Tumour rejection antigens of chemically induced sarcomas of inbred mice. *Proc. Natl Acad. Sci. USA*, **83**, 3407–3411.

SRIVASTAVA PK AND OLD LJ. (1988). Individually distinct transplantation antigens of chemically induced mouse tumours. *Immunol. Today*, **9**, 78–83.

SRIVASTAVA PK AND HEIKE M. (1991). Tumor specific immunogenicity of stress induced proteins: Convergence of two evolutionary pathways of antigen presentation. *Semin. Immunol.*, **3**, 57–66.

SRIVASTAVA PK, UDONO H, BLACHERE E AND LI Z. (1994). Heat shock protein transfer peptides during antigen processing and CTL priming. *Immunogenetics*, **39**, 93–98.

TANDON AK, CLARK GM, CHAMNES GC, FUQUA SAW, WELCH WJ, RIEHL RM AND MCGUIRE WL. (1991). Heat shock/stress response proteins: biological and clinical significance in breast cancer. *Proc. Am. Soc. Clin. Oncol.*, **9**, 23.

TETU B, LACASSE B, BOUCHARD H-L, LAGACE R, HUOT J AND LAUDRY J. (1992). Prognostic influence of HSP27 expression in malignant fibrous histocytoma: A clinicopathological and immunohistochemical study. *Cancer Res.*, **52**, 2325–2328.

THIELE CJ, REYNOLDS CP AND ISRAEL MA. (1985). Decreased expression of N-myc precedes retinopic acid induced morphological differentiation of human neuroblastoma. *Nature*, **313**, 404–406.

THOR A, BENZ C, MOORE D, GOLDMAN E, EDGERTON S, LANDRY J, SCHWARTZ L, MAYALL B, HICKEY E AND WEBER LA. (1991). Stress response protein (srp-27) determination in primary human breast carcinomas: clinical, histologic, and prognostic correlations. *J. Natl Cancer Inst.*, **83**, 154-155.

TISSIÈRES A, MITCHELL HK AND TRACEY UM. (1974). Protein synthesis in salivary glands of *D. melanogaster*. Relation to chromosome puffs. *J. Mol. Biol.*, **84**, 389-398.

TSUBOI N, ISHIKAWA M, TAMURA Y, TAKAYAMA S, TOBIOKA H, MATSUURA A, HIRAYOSHI K, NAGATA K, SATO N AND KIKUCHI K. (1994). Monoclonal antibody specifically reacting against 73-kilodalton heat shock cognate protein: possible expression on mammalian cell surface. *Hybridoma*, **13**, 373-381.

UDONO H AND SRIVASTAVA PK. (1993). Heat shock protein 70-associated peptides elicit specific cancer immunity. *J. Exp. Med.*, **178**, 1391-1396.

ULLRICH SJ, ROBINSON EA, LAIN LW, WILLINGHAM M AND APPELLA E. (1986). A mouse tumor specific transplantation antigen is a heat shock related protein. *Proc. Natl Acad. Sci. USA*, **83**, 3121-3125.

UNGAR DR, HAILAT N, STRAHLER JR, KUICK RD, GARRETT MB, SEEGER RC, REYNOLDS CP AND HANASH SM. (1994). Hsp27 expression in neuroblastoma: correlation with disease stage. *J. Natl Cancer Inst.*, **86**, 780-784.

VAN BUSKIRK A, CRUMP BL, MARGOLIASH E AND PIERCE SK. (1989). A peptide binding protein having a role in antigen presentation is a member of the hsp70 gene family. *J. Exp. Med.*, **170** (6) 1968-1970.

VOJTESEK B, KOVARIK J, DOLEZALOVA H, NENUTIL R, HAVLIS P, BRENTANI RR AND LANE DP. (1995). Absence of P53 autoantibodies in a significant proportion of breast cancer patients. *Br. J. Cancer*, **71**, 1253-1256.

YOSHINO I, GOEDEGEBUURE PS, PEOPLES GE, LEE KY AND EBERLEIN TJ. (1994). Human tumor-infiltrating $CD4^+$ T cells react to B cell lines expressing heat shock protein 70. *J. Immunol.*, **153**, 4149-4158.